# Assessment of CafA Targeted BAR-Encapsulated Nanoparticles against Oral Biofilms

**DOI:** 10.3390/pharmaceutics12090835

**Published:** 2020-09-01

**Authors:** Hetal Desai, Mohamed Y. Mahmoud, Jinlian Tan, Farnaz Minooei, Donald R. Demuth, Jill M. Steinbach-Rankins

**Affiliations:** 1 Department of Oral Immunology and Infectious Diseases, University of Louisville School of Dentistry, Louisville, KY 40202, USA; hetal.desai@louisville.edu (H.D.); jinlian.tan@louisville.edu (J.T.); 2 Department of Microbiology and Immunology, University of Louisville School of Medicine, Louisville, KY 40202, USA; 3 Department of Pharmacology and Toxicology, University of Louisville School of Medicine, Louisville, KY 40202, USA; myabde01@louisville.edu; 4 Center for Predictive Medicine, University of Louisville, Louisville, KY 40202, USA; farnaz.minooei@louisville.edu; 5 Department of Toxicology, Forensic Medicine and Veterinary Regulations, Faculty of Veterinary Medicine, Cairo University, Giza 12211, Egypt; 6 Department of Chemical Engineering, University of Louisville Speed School of Engineering, Louisville, KY 40202, USA; 7 Department of Bioengineering, University of Louisville Speed School of Engineering, Louisville, KY 40202, USA

**Keywords:** periodontal disease, *Porphyromonas gingivalis*, *Streptococcus gordonii*, oral biofilm, nanoparticles, peptide delivery, CafA protein, oral delivery, targeted oral therapy

## Abstract

*Porphyromonas gingivalis* adherence to *Streptococcus gordonii* is a crucial initial event that facilitates the colonization of *P. gingivalis*, a key pathogen in periodontal disease. As such, blocking these early interactions may present a potential avenue to limit *P. gingivalis* colonization. Nanoparticles encapsulating a synthetic peptide BAR (BAR-encapsulated NPs) inhibit *P. gingivalis*/*S. gordonii* biofilm formation 1.8-fold more potently relative to free BAR. However, BAR-encapsulated NPs, like many orally delivered formulations, may benefit from a strategy that improves their retention in an open flow environment. Here, we sought to enhance the efficacy of BAR-encapsulated NPs by modifying their surfaces with coaggregation factor A (CafA), a fimbrial protein expressed by the early colonizer, *Actinomyces oris*. We demonstrate that the targeting moiety, CafA, enhances NP binding and exhibits specificity of adherence to *S. gordonii*, relative to other oral bacterial species. Furthermore, CafA-modified NPs release inhibitory concentrations of BAR for 12 h, a time frame relevant to oral dosage form delivery. Lastly, CafA-modified NPs potently inhibit *P. gingivalis*/*S. gordonii* biofilm formation for up to 12 h and are non-toxic at therapeutically-relevant concentrations. These results suggest that CafA-modified NPs represent a novel and efficacious delivery vehicle for localized, targeted delivery of BAR to *P. gingivalis* preferred niches.

## 1. Introduction

Periodontitis is the inflammation of tooth-supporting structures, caused by dental plaque, which leads to the destruction of periodontal ligament and alveolar bone. From 2009 to 2014, 42% of adults (age > 30 years) in the United States, were diagnosed with periodontal disease. Of these patients, 7.8% had advanced periodontitis [1]. In addition to its localized oral effects, periodontal disease has been associated with systemic diseases, such as cardiovascular disease, type 2 diabetes mellitus, low birth weights, and osteoporosis [2]. Hence, along with its impact on oral structures, periodontitis constitutes a risk factor for several systemic conditions.

The current standards for periodontitis treatment involve mechanical removal of plaque/calculus by scaling and root planing, along with adjunct administration of antibiotics to prevent reoccurrence [3,4,5,6]. However, antibiotic effectiveness in the treatment of periodontitis is often limited, due to compromised penetration of antibiotics through the oral biofilm, increased drug resistance of the biofilm relative to planktonic bacteria, reduced cellular activity of bacteria within the biofilm, and prevalence of resistant pathogens in the subgingival microflora [7,8]. In a recent study, 22% to 25% of *P. gingivalis* isolates from patients with periodontitis were found to be resistant to amoxicillin, clindamycin, and metronidazole [9]. In addition, long-term, indiscriminate use of antibiotics in the treatment of periodontal disease may lead to adverse side effects such as toxicity, allergies, alteration of gut microflora, and increased antimicrobial resistance [10]. Therefore, there is a compelling need to develop novel, targeted, therapeutic approaches beyond antibiotics for the prevention and treatment of periodontal disease.

Our current understanding of periodontal disease is based on the polymicrobial synergy and dysbiosis model which proposes that periodontitis is caused by the dysbiosis of the host microbiome, induced by *Porphyromonas gingivalis* [11]. *P. gingivalis* modulates the host immune response to facilitate a shift in microbial composition to a more disruptive anaerobic community, along with an overall increase in bacterial load [12]. The altered microbiota triggers an uncontrolled host inflammatory response leading to periodontal tissue destruction [13]. *P. gingivalis* has also been shown to alter the gene expression of the microbiome [14], playing a key role in promoting dysbiosis and elevating host-microbiome virulence. Therefore, inhibiting the colonization of *P. gingivalis* presents a potential avenue to limit periodontitis and subsequent periodontal disease progression.

**Figure 1:**
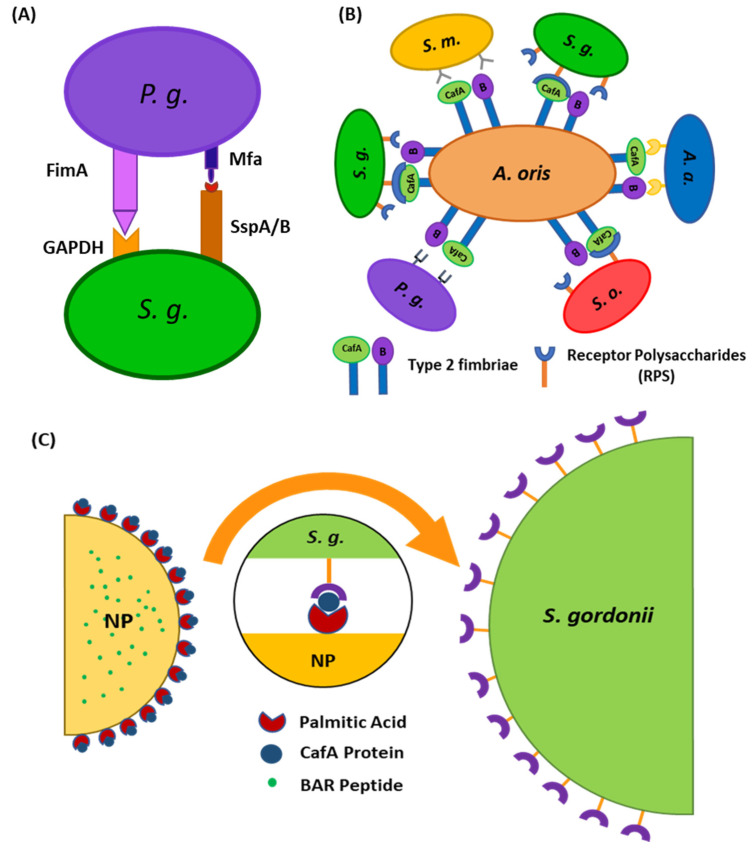
(**A**) Schematic representation of *P. gingivalis−S. gordonii* adhesion mediated via the interaction of major (fimA) and minor fimbriae (Mfa1) of *P. gingivalis* with glyceraldehyde 3-phosphate dehydrogenase (GAPDH) and motifs on SspA/B proteins of *S. gordonii*, respectively. (**B**) Schematic representing the specificity of adherence of coaggregation factor A (CafA). CafA is the tip fimbrillin of the type 2 fimbriae of *A. oris* and mediates the coaggregation of *A. oris* with commensal receptor polysaccharide (RPS) expressing oral streptococci. (**C**) Surface modification of nanoparticles with CafA protein will aid in directing nanoparticles to *S. gordonii* for targeted delivery of BAR peptide.

The adherence of *P. gingivalis* to commensal oral streptococci such as *S. gordonii*, *S. oralis*, and *S. sanguinus* is an important initial step in the colonization of *P. gingivalis* in the supragingival environment [15], after which *P. gingivalis* disperse and colonize in their preferred niches of the anaerobic subgingival pockets [16]. The initial adhesion of *P. gingivalis* to streptococci, e.g., *S. gordonii* in the supragingival environment is mediated via the interaction of the major (fimA) and minor fimbriae (Mfa1) of *P. gingivalis* with glyceraldehyde 3-phosphate dehydrogenase (GAPDH) and motifs on SspA/B proteins of *S. gordonii*, respectively [17] (Figure 1A). Blocking these initial *P. gingivalis/S. gordonii* interactions may limit the supragingival colonization of *P. gingivalis* and subsequent subgingival biofilm formation, providing ideal “early-stage” targets for therapeutic intervention. 

Previous work in our groups has demonstrated that a synthetic peptide, consisting of residues 1167 to 1193 of the surface protein SspB of *S. gordonii* designated BAR (SspB Adherence Region) blocks the Mfa1-SspB interaction and reduces the virulence of *P. gingivalis* in murine models of periodontitis [18,19,20]. However, free peptides are transiently retained in the oral cavity owing to the constant flow of saliva. Moreover, while BAR potently inhibited the initial adherence of *P. gingivalis* to *S. gordonii,* it was less effective in treating pre-established *P. gingivalis* biofilms. To address these challenges, our groups developed poly(lactic-*co*-glycolic acid) (PLGA) nanoparticles (NPs) that encapsulated BAR (BAR-encapsulated NPs) and NPs that were surface-modified with BAR (BAR-modified NPs). BAR-encapsulated NPs inhibited *P. gingivalis/S. gordonii* biofilm formation (IC_50_ = 0.7 µM) and disrupted pre-established biofilms more potently relative to free BAR [21]. Similarly, BAR-modified NPs delivered a high localized concentration of BAR peptide and improved its effectiveness relative to free BAR, through multivalent interactions with *P. gingivalis*, in in vitro and murine periodontitis models [22,23]. However, similar to free peptide, NP delivery vehicles may exhibit low retention in an open flow environment such as the oral cavity, necessitating administration of higher, more frequent doses. Furthermore, due to the time frame of peptide release (over the course of hours) from BAR-encapsulated NPs, we hypothesized that BAR-encapsulated NPs may benefit from a strategy that augments their retention in an open flow environment [21]. 

Several approaches have been employed to improve NP retention, including alteration of properties such as surface charge and hydrophobicity, and functionalizing NPs with non-specific mucoadhesive or specific targeting ligands that bind to host receptors [24]. Non-specific targeting strategies have involved integrating carboxymethyl cellulose (CMC) [25], chitosan [26], polyacrylic acid (Carbopol) [27], polyethylene glycol (PEG) [28,29], polyvinyl alcohol (PVA) [30] or polyvinyl pyrrolidone (PVP) [30,31] on NP surfaces, to improve NP retention and to act as drug depots at target sites, releasing active agent in a controlled and sustained manner [32].

Although non-specific targeting strategies have been effective, the use of specific targeting ligands has been shown to increase NP binding efficiency by two to four-fold at target sites, due to multivalent interactions with host cell receptors [33,34,35]. Several biological ligands have been used to functionalize NPs including peptides, small molecules, proteins, antibodies, and aptamers to promote target-specific delivery of therapeutic agents [36]. These ligands seek to minimize non-specific interactions between NPs and non-target cells to reduce the indiscriminate distribution of active agents at non-target sites and promote localized delivery to target sites, thereby enhancing NP efficacy [36,37]. Often, ligands are directed to exploit the endogenous differences between normal and pathological tissues and to direct therapy to classical or diseased target site markers. As one example in periodontitis, inflamed gingival epithelial cells express a higher level of the β_1_ integrin including α_2_β_1,_ α_3_β_1,_ α_5_β_1_, and α_6_β_1_, relative to normal epithelial cells. Surface modification of NPs with a peptide, RGD, which binds to the β_1_ integrin, helps to facilitate NP adherence and retention at periodontal disease sites for a prolonged duration. As such, minocycline-loaded polyethylene glycol-polylactic acid (PEG-PLA) NPs functionalized with RGD peptides demonstrated potent anti-periodontitis activity relative to non-targeted NPs and free minocycline [38]. Additionally, RGD-modified minocycline NPs delivered a higher localized concentration of minocycline to the gingiva and retained the effective concentration for a longer duration, relative to unmodified minocycline NPs [38]. This and other studies demonstrate the utility of specific ligand functionalization for periodontitis [38] or other applications [39,40,41,42].

Similar to these approaches, we sought to direct PLGA NPs to niches of the oral cavity harboring *S. gordonii*. We utilized coaggregation factor A (CafA), a fimbrial protein expressed by *Actinomyces oris*, which constitutes the tip fimbrillin of the type 2 fimbriae of *A. oris*, as a targeting ligand, to functionalize BAR-encapsulated NPs. During plaque formation, CafA binds to the GalNAcβ1-3Gal motif of the receptor polysaccharides (RPS) found on certain species of oral commensal streptococci such as *S. gordonii* and *S. oralis*, facilitating the coaggregation of actinomyces and streptococci (Figure 1B). Since CafA is the key adhesin that mediates actinomyces−streptococci binding [43], we anticipated that the functionalization of NPs with CafA would enable us to actively target NPs to areas of the oral cavity harboring commensal streptococci, to gradually release BAR in *P. gingivalis* preferred niches (Figure 1C). We hypothesized that modifying the surface of BAR-encapsulated NPs with CafA would enhance NP efficacy by augmenting their adhesion to commensal streptococci, facilitating enhanced retention for durations relevant to oral delivery (~8 to 12 h), promoting the more gradual release of BAR peptide, and resulting in potent inhibition of *P. gingivalis* adhesion to *S. gordonii* in a dual-species biofilm. Long-term it is envisioned that these NPs may be incorporated into oral healthcare products such as gels, which are traditionally used twice daily. Therefore, ideally, we seek to formulate NPs that will be retained within the oral cavity and release inhibitory concentrations of BAR peptide for a minimum of 8 to 12 h.

## 2. Materials and Methods

### 2.1. CafA Expression and Purification

CafA synthesis was done by isolating the genomic DNA of *Actinomyces oris* (ATCC^®^ 43146™, Manassas, VA, USA) from 10 mL of an overnight culture using the Wizard Genomic DNA purification kit (Promega, Madison, WI, USA) as specified by the manufacturer. The *cafA* gene was amplified by PCR using 200 ng of genomic DNA as the template and 30 pmol each of the following primers: Forward: 5′-AAG GAT CCC TGA GGC CGT TCA-3′; Reverse: 5′-CCG GAA TTC TAC GAC TTG CGG TTG GAG-3′. PCR amplification was conducted by denaturation at 94 °C for 2 min, annealing of primers and template at 63 °C for 30 s, strand extension at 72 °C for 2 min 45 s for 30 cycles, followed by a final extension cycle at 72 °C for 5 min.

The PCR product was subsequently electrophoresed in 1% agarose at 90 V for 40 min and the *cafA* band was excised and purified using the gel purification kit (Qiagen, Germantown, MD, USA). The purified *cafA* DNA (1 µg) and a sample of the pGEX-6p-1 expression vector (0.5 µg) were digested with *Bam*HI and *Eco*RI overnight at 37 °C. Prior to ligation, 50 µL of the digested vector were dephosphorylated with 4 µL calf intestinal alkaline phosphatases (New England Biolabs^®^ Inc. (NEB), Ipswich, MA, USA) at 37 °C for 30 min. Subsequently, 3 µL of protease K were added and incubated for 30 min at 50 °C to terminate the reaction. The vector and *cafA* fragments were purified using the DNA clean and concentrator kit (Zymo Research, Irvine, CA, USA) and ligated with T4 ligase (NEB). Ligation reactions were conducted according to the protocol provided by the manufacturer. Briefly, 5 μL *cafA* (80 ng), 3 μL pGEX-6p-1 (28 ng), 1 μL ligase buffer, and 1 μL T4 ligase were mixed and the ligation was carried out overnight at room temperature.

The ligation mixture was initially transformed into *E. coli* Top10. Fifty µL of competent *E. coli* Top10 were incubated with 5 µL of ligation mixture on ice for 30 min, then the sample was heat-shocked at 42 °C for 45 s and placed on ice for 2 min. Two hundred µL of SOC media were added, the sample was incubated at 37 °C for 1 h and plated on LB agar. After overnight incubation at 37 °C, single colonies were selected and cultured in 5 mL LB broth supplemented with 100 µg ampicillin. Plasmid purification was carried out using the miniprep kit (Qiagen) and the *cafA* insert was excised and confirmed by sequencing.

For CafA expression, the purified *cafA* plasmid was transformed into *E. coli* BL21 using the transformation protocol described above. After selecting and confirming the appropriate transformant, 400 mL of LB broth was inoculated with 10 mL of overnight culture and incubated to an optical density at 600 nm (O.D._600_) of 0.5. Protein expression was induced by the addition of 0.5 mM IPTG and the culture was then incubated at 18 °C for 17 h. After centrifugation at 4250× *g*, the cell pellet was suspended in 40 mL 50 mM Tris, 100 mM NaCl, 1 mg/mL lysozome, 10 µg/mL DNase I, 400 µL protease inhibitor cocktail, 10 mM CHAPS, and incubated overnight at 4 °C, followed by an additional 2 h at 25 °C. The cell suspension was then sonicated for 2 min on ice.

CafA purification was carried out with the Pierce GST Spin Purification Kit (Thermo Fisher, Waltham, MA, USA). Seventeen mL of crude cell lysate were bound to the GST column for 2 h at room temperature and the column was then centrifuged to remove unbound protein according to the specifications of the manufacturer. After washing the column with a loading buffer, the GST tag was cleaved by the addition of 50 µL precision protease (GE Health, Chicago, IL, USA) and overnight incubation at 4 °C. Released CafA was then collected by centrifugation. The sample was then sequentially dialyzed against 30 mM, 20 mM, and 10 mM Tris for 2 h each. CafA purity was determined by PAGE gels and protein concentration was determined using the BCA assay (Pierce, Waltham, MA, USA).

### 2.2. Peptide Synthesis

BAR peptide consists of residues 1167 to 1193 of the SspB surface protein expressed by *S. gordonii* and is composed of the following amino acid sequence: 

NH_2_-LEAAPKKVQDLLKKANITVKGAFQLFS-COOH. To visualize and quantify the release of the peptide from CafA-modified NPs, 6-carboxyfluorescein was covalently attached to the ε–amine of the lysine residue (underlined above) in the peptide sequence above to produce fluorescent BAR (F-BAR). Functional studies of CafA-modified NPs were carried out using NPs encapsulating unlabeled BAR peptide. Both labeled and unlabeled BAR peptide were synthesized by Biosynthesis, Inc. (Lewisville, TX, USA) and were obtained with greater than 94% purity.

### 2.3. Growth of Bacterial Strains

*Porphyromonas gingivalis* ATCC 33277 was cultured in Trypticase soy broth (TSBY) media (Difco Laboratories Inc., Livonia, MI, USA) supplemented with 0.5% (*w*/*v*) yeast extract, 1 μg/mL menadione, and 5 μg/mL hemin. The growth medium was reduced for 24 h in an anaerobic chamber (10% CO_2_, 10% H_2,_ and 80% N_2_). Twenty mL of reduced media were subsequently inoculated with 2 mL of an overnight *P. gingivalis* culture and incubated under anaerobic conditions for 48 h at 37 °C. Strains including *S. gordonii* DL-1, *S. oralis* SO34, *S. mutans* KPSP2, and *A. actinomycetemcomitans* 652 were cultured aerobically without shaking in brain-heart infusion (BHI) broth supplemented with 1% yeast extract for 16 h at 37 °C.

### 2.4. Conjugation of CafA Protein with Palmitic Acid

CafA-palmitate was synthesized as previously described [44,45,46]. Briefly, 2 mg of purified CafA was dissolved in 1.2 mL of 2% (*w*/*v*) sodium deoxycholate (NaDC) in phosphate-buffered saline (137 mM NaCl, 2.7 mM KCl, 1.8 mM KH_2_PO_4_, 10 mM Na_2_HPO_4_; PBS; pH = 7.4) and warmed to 37 °C. Next, a solution with a 14-fold molar excess of the palmitic acid-*N*-hydroxysuccinimide ester (NHS-palmitic acid; Sigma-Aldrich, St Louis, MO, USA) was prepared by dissolving NHS-palmitic acid in 2% (*w*/*v*) NaDC at 0.125 mg/mL. The solution was sonicated until well mixed in an ultrasonic bath and 800 µL of this solution was added dropwise to the reaction vial containing CafA and reacted overnight at 37 °C. To remove excess fatty acid and hydrolyzed ester, reactants were dialyzed against 1.2 L of PBS with 0.15% deoxycholate, using a 3500 molecular weight cut-off dialysis tube. After overnight dialysis at 37 °C, CafA-palmitate was stored at 4 °C until use.

### 2.5. Synthesis of CafA-Modified Blank NPs

Nanoparticles were synthesized using poly(lactic-*co*-glycolic acid) (PLGA) carboxyl-terminated polymer (0.55–0.75 dL/g inherent viscosity; LACTEL^®^). To formulate CafA-modified nanoparticles, a previously described oil-in-water (*o/w*) single emulsion technique was used [47,48]. Briefly, 100 mg PLGA was dissolved in 2 mL dichloromethane (DCM) by overnight incubation at 25 °C. The next day, 2 mL of 5% (*w*/*v*) polyvinyl alcohol (PVA) was added to 2 mL CafA-palmitate solution. This solution was vortexed and 2 mL of PLGA/DCM solution was added to it in a dropwise manner. The resulting solution was ultrasonicated and excess DCM was evaporated by adding the solution to 50 mL of 0.3% (*w*/*v*) PVA and mixing using a magnetic stir bar for 3 h. After evaporation, the NP solution was centrifuged at 13,000 rpm (20,442× *g*) at 4 °C for 10 min. The supernatant, containing unreacted CafA conjugates was discarded, and the NPs were washed twice with deionized water (diH_2_O) followed by centrifugation at 13,000 rpm (20,442× *g*) at 4 °C for 10 min. After washing, CafA-modified NPs were suspended in 5 mL of diH_2_O, freeze-dried at −80 °C, and lyophilized under 10 mTorr for 48 h.

### 2.6. Synthesis of CafA-Modified NPs Encapsulating C6/F-BAR/BAR

For our experiments, three different types of CafA-modified NPs were synthesized. CafA-modified NPs encapsulating the fluorescent dye, Coumarin 6 (C6), were synthesized to assess the binding functionality of CafA surface modification. CafA-modified NPs encapsulating fluorescent BAR (F-BAR) were synthesized to determine the loading and controlled release characteristics of BAR from surface-modified NPs, and CafA-modified NPs encapsulating unlabeled BAR were synthesized to determine the efficacy of NP-mediated inhibition of *P. gingivalis* adherence to streptococci using a two-species biofilm model.

CafA-modified NPs encapsulating C6 or BAR were synthesized similar to above using a previously described *w/o/w* double emulsion solvent evaporation technique [45,46]. Briefly, C6 was dissolved overnight in 200 μL DCM at a concentration of 15 μg/mg PLGA. In parallel, 100 mg of PLGA crystals were dissolved in 2 mL of DCM by overnight incubation at 25 °C. The following day, the C6 DCM solution was first emulsified in the PLGA/DCM solution by vortexing, followed by ultrasonication to achieve a homogeneous suspension. Next, the homogeneous suspension was added dropwise to a mixture of 2 mL of 5% (*w*/*v*) polyvinyl alcohol (PVA) and 2 mL CafA-palmitate solution while vortexing, followed by ultrasonication. Excess DCM was evaporated and NPs were collected as described above. CafA-modified NPs encapsulating F-BAR/BAR were synthesized using a similar approach. All synthesis reactions were protected from exposure to light. For the synthesis of CafA-modified NPs encapsulating either F-BAR or unlabeled BAR, the peptide was dissolved in 200 μL Tris EDTA buffer (VWR; 100 mM Tris HCl,10 mM EDTA at a pH of 8.0; TE buffer) at a concentration of 43 µg BAR/mg PLGA [21].

### 2.7. NP Characterization: NP Morphology, Size, and Zeta Potential

Unhydrated NP morphology, diameter, and size distribution were determined using scanning electron microscopy (SEM, XL-30 ESEM-FEG SEM, FEI Company, Hillsboro, OR, USA). Lyophilized NPs were mounted on carbon tape and sputter-coated with a thin layer of gold/palladium. Average diameters of 500 particles were determined from SEM images (*n* = 3, 50 NPs measured per field of view) using image analysis software (ImageJ, National Institutes of Health, version 1.5a, ImageJ.nih.gov, Bethesda, MD, USA). The zeta potential was measured to determine the surface charge of hydrated NPs. Briefly, 1 mg/mL samples of unmodified PLGA NPs and CafA-modified NPs in diH_2_O were prepared. After vortexing and sonication, samples were diluted at a 1:10 ratio in diH_2_O. One mL was aliquoted to the cuvette for analysis using Brookhaven Instrument Corporation 90 plus.

### 2.8. Surface Density of CafA

CafA-modified NPs were synthesized using varying input concentrations of CafA protein (5 to 80 µg/mg polymer). For each input condition, the resulting concentration of the CafA conjugated to the NP surface was measured using the microBCA assay (Pierce, Waltham, MA, USA). CafA-modified NPs (1 mg) were suspended in 1% dimethyl sulfoxide (DMSO, Sigma Aldrich, St. Louis, MO, USA) in PBS. Aliquots (100 µL) of the NP samples were analyzed in triplicate in a microtiter plate and NP-associated absorbance was measured by spectrophotometry at a wavelength of 562 nm. The concentration of the CafA was determined by comparing absorbance values to a known standard curve of CafA and subtracting the background absorbance values of unmodified NPs (control group).

### 2.9. Loading and Release Kinetics of BAR Peptide from Unmodified and CafA-Modified NPs

Nanoparticles modified with an intermediate density of CafA (20 µg/mg polymer) were selected for subsequent characterization and functionality studies. To determine BAR peptide loading, approximately 2 mg CafA-modified NPs encapsulating F-BAR were dissolved in 1 mL DMSO. Aliquots (100 µL) of the NP samples were analyzed in triplicate in a microtiter plate and the amount of F-BAR in the dissolved solution was determined by measuring fluorescence (488/518 nm excitation/emission) and quantified by comparing these values to a known standard curve of F-BAR.

To analyze the release kinetics of F-BAR, aliquots of ~1 to 3 mg CafA-modified and unmodified NPs encapsulating F-BAR were incubated in microcentrifuge tubes containing 1 mL PBS (pH 7.4) at 37 °C with gentle horizontal agitation. At fixed time points (1, 2, 4, 8, and 24 h) after the initial suspension, the samples were centrifuged at 18,900× *g* and the supernatant was collected. The pelleted NPs were then suspended in fresh PBS and incubated until the next time point. The amount of F-BAR in the supernatant was determined by measuring fluorescence (488/518 nm excitation/emission) and quantified by comparing these values to a known standard curve of F-BAR.

### 2.10. Determination of Surface Modification Functionality

#### 2.10.1. Duration of CafA-Modified NP Adhesion

The functionality of surface modification and preservation of the function of CafA protein during NP synthesis was analyzed using two approaches. To determine the duration of retention of CafA-modified C6 NPs on *S. gordonii* cells, *S. gordonii* was cultured as previously described and bacterial cells were harvested by centrifuging 10 mL of culture at 3700× *g* for 5 min. The supernatant was discarded and the pelleted cells were suspended in 1 mL of PBS. The O.D. at 600 nm of the cell suspension was adjusted to 0.2 and 100 µL of the *S. gordonii* cell suspension was added to each well of a 96-well microtiter plate and incubated overnight at 4 °C. After removing unbound cells, the wells were blocked for non-specific binding with 300 µL of 0.3% bovine serum albumin (BSA) for 1 h. Thereafter, the microtiter plate was washed three times with 1× PBS containing 0.05% Tween (PBST). Immobilized *S. gordonii* cells were then incubated with 100 µL of CafA-modified C6 NPs (0.25 mg/mL), unmodified C6 NPs (0.25 mg/mL, control group), or PBST in the absence of NPs in triplicate for 1 h on a rocker platform. After washing three times with PBST, the cell-associated fluorescence was measured using Synergy HT reader (BioTek, Winooski, VT, USA) (485/520 nm excitation/emission). After subtracting the control fluorescence (*S. gordonii* incubated with PBST), the initial reading (at *t* = 0) was defined as 100% binding. After obtaining the initial reading, 100 µL of PBST was added to each well and at fixed time points (1, 2, 4, 8, 12 h), the PBST was removed and the cell-associated fluorescence that remained was measured. Subsequently, an additional 100 µL aliquot of fresh PBST was added per well and incubated until the next time point was reached.

#### 2.10.2. Specificity of CafA-Modified NP Adhesion

To determine the specificity of CafA adhesion, the adherence of CafA-modified C6 NPs to *S. gordonii* DL-1, *S. oralis* SO34, *S. mutans* KPSP2, *P. gingivalis* ATCC 33277, and *A. actinomycetemcomitans* 652 cells was measured. CafA binds to the GalNAcβ1-3Gal motif of the receptor polysaccharides (RPS) found only on commensal oral streptococci such as *S. gordonii* and *S. oralis*. It does not bind to bacteria lacking this motif and therefore, *S. mutans*, *P. gingivalis,* and *A. actinomycetemcomitans* were selected as negative controls. Each of the organisms was cultured and harvested as described previously, and the final O.D. at 600 nm for each cell suspension was adjusted to 0.2. The bacterial cells were immobilized on a 96-well microtiter plate as described above and after overnight incubation, wells were blocked for non-specific binding with 300 µL of 3% bovine serum albumin (BSA) for 1 h. The plate was washed three times with PBST, and immobilized bacterial cells were incubated with 100 µL of CafA-modified C6 NPs (0.25 mg/mL), unmodified C6 NPs (0.25 mg/mL) or with PBST in triplicate for 1 h on a rocker platform. The microtiter plate was again washed three times with PBST, and cell-associated fluorescence was measured (485/520 nm excitation/emission). To determine the final cell-associated fluorescence, the readings obtained from bacteria incubated with unmodified C6 NPs (control group) were subtracted from that of bacteria incubated with CafA-modified C6 NPs. Data were analyzed using an independent *t*-test.

### 2.11. CafA-Modified NP-Mediated Inhibition of P. gingivalis Adherence to Streptococci

*S. gordonii* DL-1 was cultured as previously described and bacterial cells were harvested by centrifuging 10 mL of culture at 3700× *g* for 5 min. The supernatant was discarded, and the pelleted cells were suspended in 1 mL of 1× PBS in a microcentrifuge tube. The cells were labeled with 20 µL of 10 mM hexidium iodide (Thermo Fisher Scientific, Waltham, MA, USA) for 15 min on a rocker platform at room temperature. The microcentrifuge tube was covered with foil, centrifuged at 3700× *g* for 5 min, and the pelleted cells were suspended in 1 mL of 1× PBS. The O.D. at 600 nm was measured as previously described and adjusted to 0.8. One mL of the resulting cell suspension was added to each well of a 12-well microtiter plate containing a glass coverslip. The cells were incubated overnight under anaerobic conditions on a rocker platform and protected from light.

On the following day, the wells were washed to remove unbound *S. gordonii* cells. The immobilized *S. gordonii* cells were incubated with CafA-modified BAR NPs (treatment) or CafA-modified blank NPs (control) at a concentration of 240 µg/mL for different durations (0, 2, 4, 8, 12 h) on a rocker platform. Due to the 50% inhibitory concentration (IC_50_) of free BAR peptide equivalent to 1.3 µM or ~4 µg, and NP loading results, we calculated that 240 µg CafA-modified BAR NPs would encapsulate an equivalent amount of BAR. At the initial time point (*t* = 0), CafA-modified NPs and *P. gingivalis* were added simultaneously in triplicate. At each subsequent time point (*t* = 2, 4, 8, 12 h) the supernatant containing the unbound NPs and released BAR was removed and *P. gingivalis* was added in triplicate to the control and treatment plates as described below.

*P. gingivalis* ATCC 33277 was cultured and harvested as previously described. *P. gingivalis* was labeled with 15 µL of 5-(6) carboxyfluorescein-succinyl ester (4 mg/mL) for 30 min, centrifuged at 3700× *g* for 2 min and the pelleted cells were suspended in 1 mL of 1× PBS. The O.D. at 600 nm was measured and adjusted to 0.4. At each time point, 1 mL of labeled *P. gingivalis* cell suspension (O.D 0.4) was added to the treatment and control plates in triplicate.

The plates were incubated at 37 °C for 24 h under anaerobic conditions. The subsequent day the supernatant was removed, the wells were washed with 1× PBS to remove unbound bacterial cells, and adherent cells were fixed with 4% (*w*/*v*) paraformaldehyde. The coverslips were mounted on a glass slide using clear nail polish. The prepared slides were stored at 4 °C.

The dual-species biofilms were visualized using a LEICA SP8 confocal microscope (Leica Microsystems Inc., Buffalo Grove, IL, USA) under 60× magnification. Three-dimensional z-stack biofilm images were obtained using a z-step size of 0.7 µm. Images were analyzed using Volocity software (version 6.3; Perkin Elmer, Waltham, MA, USA) to quantify the bacterial populations by quantifying fluorescence (*S. gordonii*—red, *P. gingivalis*—green). Adherence of *P. gingivalis* to streptococci was determined by measuring the green to red fluorescence ratio (GR). Inhibition at each time point was analyzed in triplicate and three independent frames were obtained for each well. The percentage of *P. gingivalis* inhibition was calculated using the formula: (1 − GR treatment/GR control) * 100 [21,22]. The mean and standard deviation of inhibition at each time point was calculated.

### 2.12. Determination of CafA-Modified and Unmodified BAR-NP In Vitro Cytotoxicity

#### 2.12.1. MTT Assay

TIGK cells were seeded in 12-well plates at a density of 6 × 10^4^ cells in 1 mL media per well and incubated for 24 h to allow for 60 to 70% confluency and sufficient adhesion. Cells were treated with 1.3 or 3.4 µM of CafA-modified BAR-NPs or unmodified BAR-NPs. After 24 h, 100 µL of MTT solution (3-(4,5-dimethylthiazol-2-yl)-2,5-diphenyltetrazolium bromide, 10% of total volume, Sigma Aldrich, St. Louis, MA, USA) was added to the media of all samples. The solution was incubated at 37 °C for 4 h. After this period, 550 µL of lysis buffer (50% of total volume, Fisher Scientific, Waltham, MA, USA) was added to the media of each well, and plates were incubated overnight. The absorbance of each well was read at 570 nm, and the sample absorbance was normalized to the absorbance of untreated cells (media only). Treatment with 10% DMSO media (100 µL DMSO in 900 µL media) was used as a positive control for cell death.

#### 2.12.2. ATP Assay

Total ATP levels in cell culture were assessed by using the CellTiter-Glo reagent (Promega, Madison, WI, USA), as described by the manufacturer. TIGK cells were seeded at a density of 6 × 10^4^ cells in 1 mL media per well and incubated at 37 °C, 5% CO_2_ for 24 h in a 12-well flat-bottom plate. Cells were then incubated with CafA-modified BAR-NPs or unmodified BAR-NPs (1.3 or 3.4 µM) for 24 h at 37 °C in 5% CO_2_. Cells were then lysed with 500 µL of 0.1% Triton X-100 for 30 min at 37 °C. The lysates were collected and centrifuged at 1000× *g* for 10 min at 4 °C, and 50 µL of supernatant was mixed with 50 µL of CellTiter-Glo reagent. Samples were incubated at ambient temperature for 10 min in a black 96-well plate in the dark. Total luminescence was measured with a Victor 3 luminometer (Perkin-Elmer, Inc., Waltham, MA, USA). Cells incubated with 1 ng of staurosporine or with medium-only served as positive and negative controls for cell death, respectively.

#### 2.12.3. LDH Assay

Cell membrane leakage was measured by the release of lactate dehydrogenase (LDH). Extracellular LDH was quantified using a CytoTox96^®^ non-radioactive cytotoxicity assay (Promega, Madison, WI, USA) as described by the manufacturer. TIGK cells were plated at a density of 6 × 10^4^ cells in 1 mL media per well in a 12-well flat-bottom plate and incubated at 37 °C, 5% CO_2_ for 24 h. CafA-modified BAR NPs or unmodified BAR NPs (1.3 or 3.4 µM) were added to cells in triplicate for 24 h at 37 °C in 5% CO_2_. Fifty microliters of supernatant from CafA-modified BAR NP- and unmodified BAR NP-treated (1.3 and 3.4 µM) cells were added to the LDH substrate and incubated at room temperature for 30 min. The reactions were subsequently terminated by adding 50 µL of stop solution. LDH activity was determined by measuring the optical density of the solution at 490 nm. Cells treated with staurosporine or with medium-only served as positive and negative controls for cell death, respectively.

Data from each of the toxicity tests were analyzed using ANOVA after passing Bartlett’s and Brown-Forsythe tests for homogeneity of variances using GraphPad InStat (La Jolla, CA, USA). A pair-wise, parametric analysis of variance using a Bonferroni multiple comparison post-test was used to determine the statistical significance between the individual groups. A *p*-value of ≤0.05 was considered to be statistically significant.

## 3. Results

### 3.1. NP Characterization: NP Morphology, Size, and Zeta Potential

**Figure 2:**
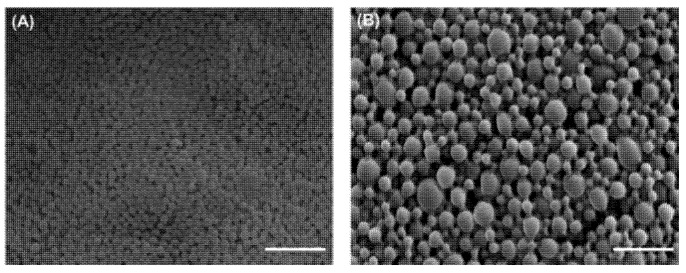
SEM images of (**A**) CafA-modified and (**B**) unmodified BAR-encapsulated PLGA NPs. Images are representative of a minimum of 3 independent samples, with *n* > 500 NPs assessed in total. Scale bars represent 1 µm.

The morphology of CafA-modified BAR NPs, relative to unmodified BAR NPs, is shown in Figure 2. CafA-modified BAR NPs demonstrated a spherical morphology and smaller size relative to unmodified BAR NPs. The average unhydrated diameters of CafA-modified BAR NPs and unmodified BAR NPs measured from SEM images were 89.7 ± 26.3 nm and 165.8 ± 33.4 nm, respectively, while the corresponding size ranges were 68.7 to 143.5 nm and 86.7 to 209.6 nm. The zeta potentials of CafA-modified NPs and unmodified NPs were −38.69 ± 0.21 and −39.58 ± 0.26, respectively. The similarity in the zeta potential of CafA-modified and unmodified NPs is attributed to the neutral charge of CafA (isoelectric potential = 7.1).

### 3.2. Quantification of CafA Surface Density

**Figure 3:**
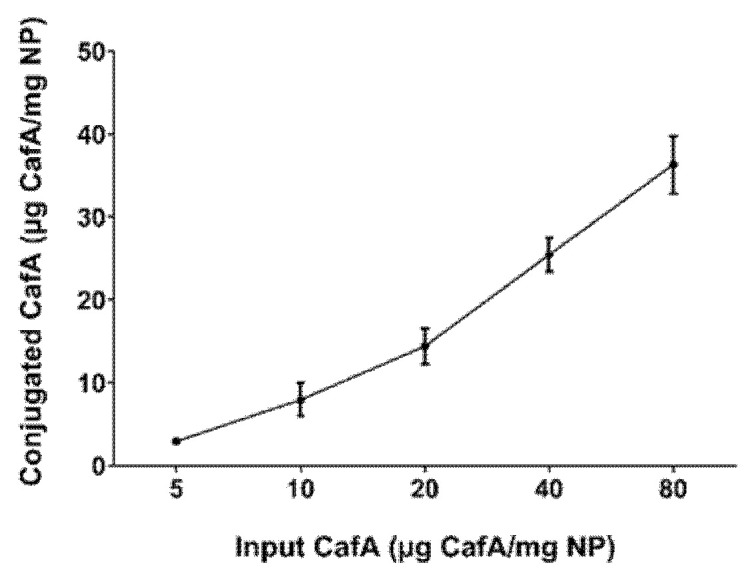
CafA surface density as a function of CafA input concentration. The total amount of CafA conjugated to the NP surface was determined using the microBCA assay. The amount of CafA conjugated to the NP surface varied directly with the input concentration of the CafA used during synthesis. Data represent the mean NP-associated CafA ± standard deviation.

The concentration of CafA conjugated to the NP surface was measured using the microBCA assay. The total protein content ranged from 3 to 36 µg CafA/mg polymer and varied directly with the input concentration of CafA (5 to 80 µg CafA/mg polymer) used during synthesis. The conjugation efficiency ranged from 45 to 79%, with higher conjugation efficiency observed at lower concentrations (Figure 3, Table 1). Although NP surface saturation was not achieved with these input concentrations, the results suggest that an increased surface density may be attained with higher CafA input.

### 3.3. Loading and Release Kinetics of BAR Peptide from Unmodified and CafA-Modified NPs

Nanoparticles modified with an intermediate density of CafA (20 µg/mg polymer) were selected for subsequent characterization and functionality studies, as they represent a practical minimum modification density that in preliminary studies (and results here) provided a therapeutically-relevant concentration of BAR release. To determine the loading of BAR peptide in CafA-modified and unmodified NPs, the amount of F-BAR from solubilized NPs was quantified by measuring fluorescence (488/518 nm excitation/emission) and comparing these values to an F-BAR standard curve. Loading experiments showed that CafA-modified and unmodified NPs encapsulated 15.73 ± 1.9 µg and 16.95 ± 0.8 of BAR per mg of NP respectively, corresponding to loading efficiencies of 37% and 39%, suggesting that CafA surface modification at this density had minimal effect on BAR loading.

**Figure 4:**
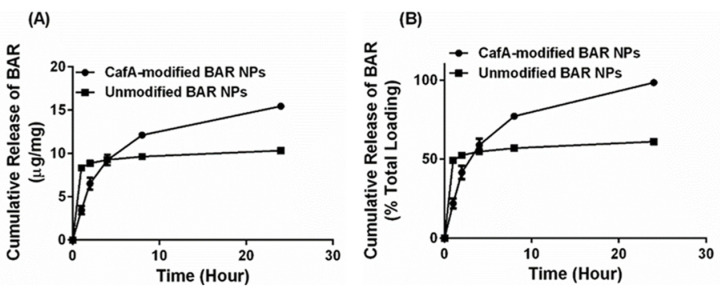
Cumulative release of BAR as a function of (**A**) mass (µg BAR per mg NP) and (**B**) percent of total BAR encapsulated. Error bars represent the mean BAR released ± standard deviation. Please note that in some cases the error bars are smaller than the data point markers.

In release experiments, sample eluates were taken 1, 2, 4, 8, and 24 h after incubation in PBS. The overall release trends showed that CafA-modified NPs demonstrated the slower release of BAR, relative to unmodified NPs; however, inhibitory concentrations of BAR peptide (2 to 4 µg/mg NP) were released from CafA-modified NPs at each of the measured time points up to 8 h. For unmodified NPs, more rapid release profiles were observed, with greater than 50% of BAR peptide released within 1 h and a plateau in the release after. After 2, 4, 8, and 24 h, less than 1 µg of peptide/mg NP was released from the unmodified NPs (Figure 4), demonstrating inadequate, non-inhibitory levels of release. In comparison, CafA-modified NPs released 23% of BAR during the first hour, and inhibitory concentrations (3.1, 2.7, 2.9, and 3.3 µg/mg) of BAR peptide after 2, 4, 8, and 24 h. Cumulatively, after 24 h, BAR peptide (15.5 µg/mg) was completely released from CafA-modified BAR NPs, whereas only 61% of the encapsulated BAR (10.3 µg/mg) was released from unmodified BAR-encapsulated NPs. Thus, after 24 h, the total quantity of BAR released from CafA-modified NPs was significantly higher than the amount of BAR released from unmodified NPs.

### 3.4. Determination of Surface Modification Functionality

#### 3.4.1. Duration of CafA-Modified NP Adhesion

**Figure 5:**
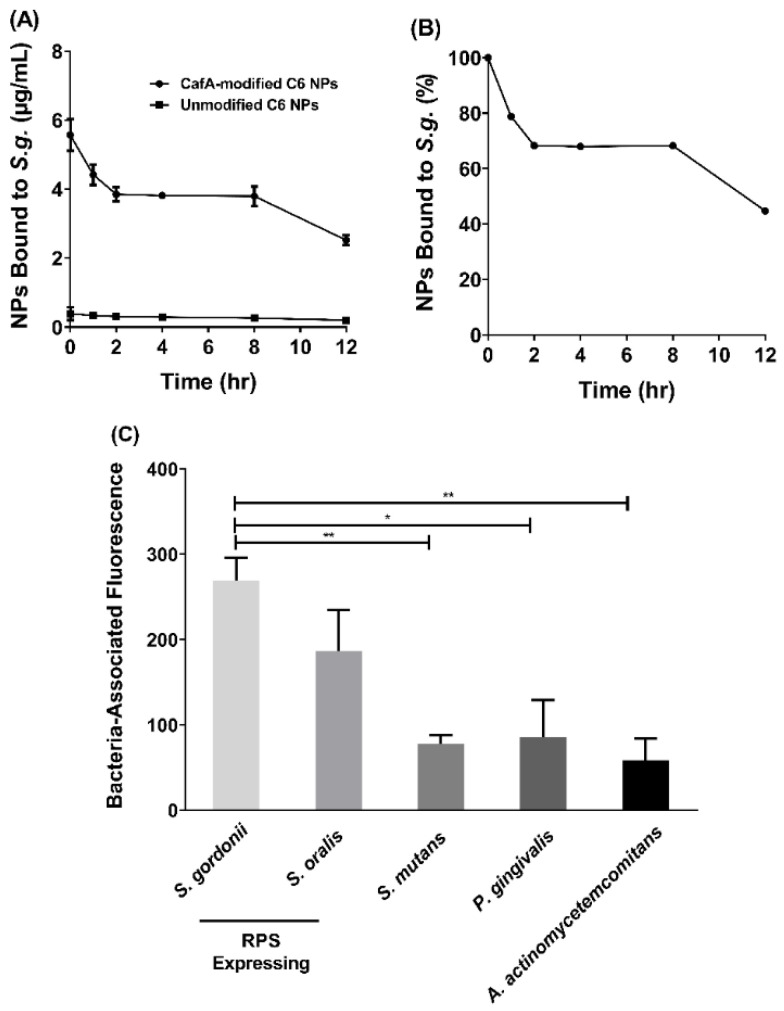
(**A**) After one hour of initial binding (*t* = 0), a 14.4-fold higher concentration of CafA-modified NPs bound to *S. g.* relative to unmodified NPs and this difference in concentration was maintained for up to 12 h. (**B**) After 12 h, 45% of the administered CafA-modified NPs remain bound to *S. g.* (**C**) CafA binds to GalNAcβ1-3Gal motif of the receptor polysaccharides found only on commensal oral streptococci such as *S. gordonii* and *S. oralis*, and demonstrates minimal binding to bacteria lacking this motif. Here, CafA-modified NPs bound to *S. gordonii* DL-1 and *S. oralis* SO34 at a higher concentration relative to *S. mutans* KPSP2, *P. gingivalis* ATCC 33277, and *A. actinomycetemcomitans* 652. Asterisks denote statistical significance between two groups (* *p* < 0.01, ** *p* < 0.001).

CafA-modified C6 NPs were administered to immobilized *S. gordonii* cells for 1 h, after which unbound NPs were washed and cell-associated fluorescence was measured, to determine the amount of NPs that initially bound to *S. gordonii* (*t* = 0). As shown in Figure 5A, at the initial time point (*t* = 0), CafA-modified NPs bound to *S. gordonii* at a 14.4-fold higher concentration (5.6 µg/mL), relative to unmodified NPs (0.4 µg/mL). After 1 h of initial binding (*t* = 1), 79% of CafA-modified NPs remain bound to *S. gordonii* and after 2 h (*t* = 2), 69% of CafA-modified NPs remain bound to *S. gordonii*, maintaining binding through 8 h. Between 8 and 12 h, an additional 23% NP dissociation was observed, resulting in 45% (2.5 µg/mL) of CafA-modified and 52% of unmodified NPs (0.2 µg/mL) associated with *S. gordonii*. After 12 h, this resulted in a 12.4-fold higher concentration of CafA-modified NPs bound to *S. gordonii* (Figure 5B). These results indicate that the ratio of CafA-modified to unmodified NPs bound to *S. gordonii* was maintained after the first wash and suggest that the targeting moiety, CafA, improves the binding efficiency of unmodified NPs and enhances their retention by significantly increasing the concentration of CafA-modified NPs that bind to *S. gordonii* relative to unmodified NPs.

#### 3.4.2. Specificity of CafA-Modified NP Adhesion

While the overall adhesion and retention of CafA-modified NPs to *S. gordonii* is important, we sought to assess the differences in CafA-modified NP binding across several bacteria to determine the specificity of NP adhesion to RPS-expressing bacteria (i.e., *S. gordonii* and *S. oralis*). The adherence of CafA-modified NPs to *S. gordonii* DL-1was measured relative to *S. oralis* SO34, *S. mutans* KPSP2, *P. gingivalis* ATCC 33277, and *A. actinomycetemcomitans* 652 cells (Figure 5C). For CafA-modified NPs, no statistical significance was observed in the bacteria-associated fluorescence between commensal oral streptococci, *S. gordonii* DL-1 and *S. oralis* SO34, that express the GalNAcβ1-3Gal motif. However, compared to non-commensal bacterial groups that lacked the CafA binding motif (*S. mutans, P. gingivalis*, and *A. actinomycetemcomitans*), *S. gordonii* DL-1 associated fluorescence was 3.5-fold higher relative to *S. mutans* KPSP2, 3.2-fold higher relative to *P. gingivalis* ATCC 33277, and 4.6-fold higher relative to *A. actinomycetemcomitans* 652. The fluorescence associated with *S. gordonii* DL-1 was found to be statistically significant (*p* ≤ 0.05) relative to *S. mutans* KPSP2, *P. gingivalis* ATCC 33277, and *A. actinomycetemcomitans* 652. No statistical significance in bacteria-associated fluorescence was observed between non-commensal bacterial groups. Together these results indicate that CafA-modified NPs bind to RPS-expressing commensal streptococci at a higher concentration relative to non-commensal organisms, demonstrating the specificity of adherence.

### 3.5. CafA-Modified NP-Mediated Inhibition of P. gingivalis Adherence to Streptococci

**Figure 6:**
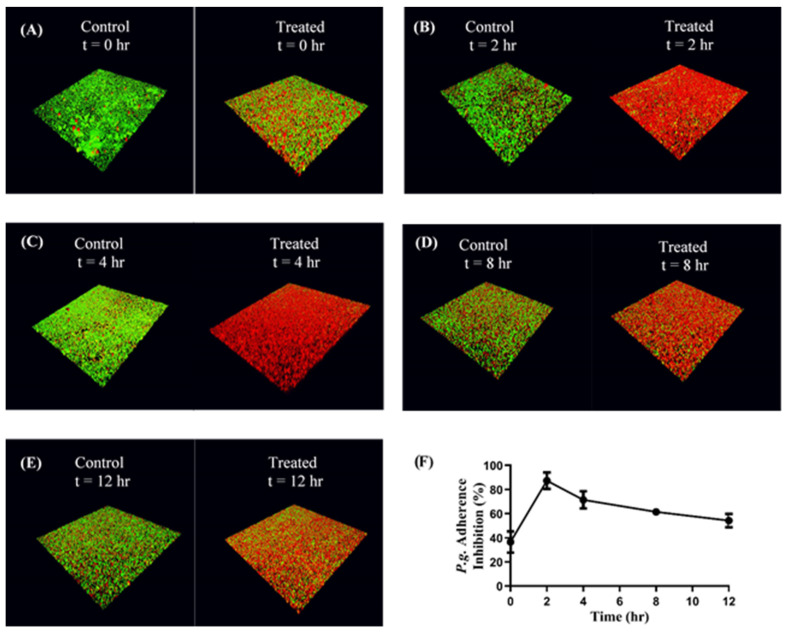
(**A**–**E**): Adherence inhibition of *P. gingivalis* to *S. gordonii* after different durations of administration. CafA-modified BAR NPs inhibited *P. gingivalis* adherence for over 12 h relative to CafA-modified blank NPs (control). Biofilms were visualized using confocal microscopy and the ratio to green *(P. g.*) to red *(S. g.*) fluorescence in z-stack images was determined using Volocity software. (**F**) CafA-modified BAR NP mediated inhibition of *P. gingivalis* adherence to *S. gordonii* at different time points in a dual-species biofilm. Error bars represent the mean inhibition ± standard deviation.

Functional inhibition assays were performed to determine the impact of CafA-modified BAR NPs on the inhibition of *P. gingivalis* adhesion to *S. gordonii*. Immobilized *S. gordonii* were incubated with CafA-modified BAR NPs (treatment group) or CafA-modified blank NPs (control) for different durations (0, 2, 4, 8, 12 h). At the initial time point (*t* = 0), CafA-modified NPs and *P. gingivalis* were added simultaneously in triplicate. At each subsequent time point (*t* = 2, 4, 8, 12 h) the supernatant containing the unbound NPs and released BAR was removed and *P. gingivalis* was added in triplicate to the control and treatment plates. The plates were incubated for 24 h and the formed biofilms were visualized using confocal microscopy. Representative images of treatment and control biofilms are shown in Figure 6A–E. At each time point (*t* = 0, 2, 4, 8, and 12 h), *P. gingivalis* adherence to *S. gordonii* was significantly reduced in the presence of CafA-modified BAR NPs, relative to control CafA-modified blank NPs (Figure 6F, Table 2). After initial administration (*t* = 0), *P. gingivalis* adherence was inhibited by 36.6 ± 8.8%. After 2 h, more than 70% inhibition was observed and maintained for up to 4 h relative to control CafA-modified blank NPs. After 8 and 12 h, time frames relevant to oral administration regimens, *P. gingivalis* binding to *S. gordonii* was inhibited by 61.5 ± 2.7% and 54.3 ± 5.6%, demonstrating the potential of CafA-modified BAR NPs to significantly inhibit *P. gingivalis* adherence to *S. gordonii*. Hence, CafA-modified NPs maintained over 50% inhibition for 12 h relative to control NPs.

### 3.6. Determination of CafA-Modified and Unmodified BAR-NP In Vitro Cytotoxicity

#### 3.6.1. MTT Assay

**Figure 7:**
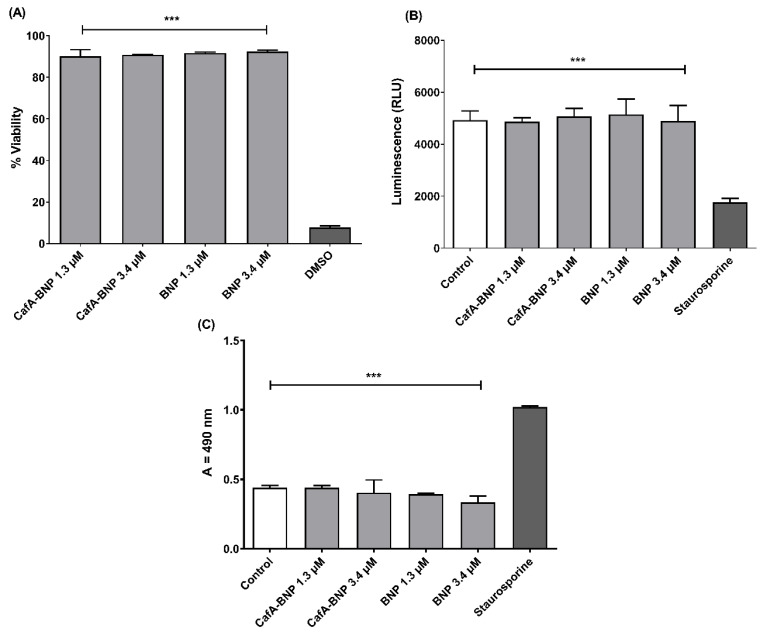
(**A**) TIGK cell viability was assessed after CafA-modified BAR NPs or unmodified BAR NPs administration. CafA-modified BAR NPs and unmodified BAR NPs were non-toxic, relative to cells treated with DMSO. (**B**) ATP levels from CafA-modified BAR NPs and unmodified BAR NPs (1.3, 3.4 µM) treated cells showed nearly the same level of ATP concentration of control cells (treated with medium only), while ATP levels in the staurosporine-treated cells were significantly lower than the control (treated with medium only), CafA-modified BAR NPs, and unmodified BAR NPs-treated cells (***, *p* ≤ 0.001). (**C**) No significant release of LDH was observed from TIGK cells treated with CafA-modified BAR NPs and unmodified BAR NPs, relative to control cells. Staurosporine-treated cells demonstrated significantly elevated LDH levels (***, *p* ≤ 0.001). Data represent the mean ± standard deviation (*n* = 3).

To assess the effect of CafA-modified BAR NPs or unmodified BAR NPs on the viability of TIGK cells, cells were incubated with CafA-modified or unmodified BAR NPs (1.3 or 3.4 µM) for 24 h and viability was measured using the MTT assay. As shown in Figure 7A, treated cells exhibited little loss in viability, suggesting CafA-modified and unmodified BAR NPs are biocompatible with TIGK cells when applied for 24 h.

#### 3.6.2. ATP Assay

Cytotoxicity was also determined by assessing the metabolic activity of TIGK cells by measuring ATP levels. As shown in Figure 7B, staurosporine-treated cells demonstrated significantly lower levels of ATP (*p* ≤ 0.001) than were observed for untreated; CafA-modified BAR NP-treated; unmodified BAR NP-treated cells.

#### 3.6.3. LDH Assay

As some peptides are known to damage the cell membrane, we next measured LDH activity as a marker for cell membrane integrity after treatment with CafA-modified or unmodified BAR NPs. Figure 7C shows that LDH levels released from cells treated with CafA-modified or unmodified BAR NPs (1.3 or 3.4 µM) were negligible when compared to control (medium-treated) cells. In contrast, LDH activity released from cells treated with staurosporine was significantly higher than control or treated cells (*p* ≤ 0.001), indicating that CafA-modified BAR NPs or unmodified BAR NPs do not compromise cell membrane integrity.

## 4. Discussion

The adherence of *P. gingivalis* to *S. gordonii* is a crucial, initial event that facilitates the colonization of *P. gingivalis* in the oral cavity. The binding of *P. gingivalis* to *S. gordonii* is driven by a protein−protein interaction between Mfa1, the structural subunit of the minor fimbriae of *P. gingivalis*, and discrete domains of the streptococcal cell surface protein, SspB, of the antigen I/II family [15,49,50,51]. These early interactions provide ideal targets for therapeutic interventions to limit the supragingival colonization of *P. gingivalis* in the oral cavity.

Previous studies have shown that a synthetic peptide designated BAR potently inhibits *P. gingivalis/S. gordonii* adherence and reduces the virulence of *P. gingivalis* in a murine model of periodontitis [18,19,20]. However, owing to the constant flow of saliva in the oral cavity, the free peptide is only transiently retained. Moreover, a higher concentration of free peptide is needed to disrupt pre-established *P. gingivalis* biofilms. To overcome this challenge, we developed polymeric nanoparticles that encapsulated BAR. BAR-encapsulated NPs (IC_50_ = 0.7 µM) were found to be more efficacious relative to the free peptide (IC_50_ = 1.3 µM) and inhibited pre-established biofilms potently. However, similar to free peptide, BAR-encapsulated NPs also lack a mechanism by which to prolong retention in the oral cavity. As BAR-encapsulated NPs release BAR peptide over the course of hours, they may benefit from a strategy to augment their retention in the oral cavity [21]. Given this, the goal of this study was to formulate NPs with improved binding to the GalNAcβ1-3Gal motif of the receptor polysaccharides (RPS) expressed by commensal oral streptococci. In parallel, we sought to prolong the release of inhibitory concentrations of BAR peptide for 8 to 12 h, for future application in oral hygiene products, such as oral gels, that are traditionally administered twice daily.

CafA is the tip fimbrillin of type 2 fimbriae of *A. oris*, and during plaque development, it mediates the coaggregation of *A. oris* with commensal oral streptococci. CafA binds to the GalNAcβ1-3Gal motif of the RPS found on commensal oral streptococci such as *S. gordonii* and *S. oralis* that promote *P. gingivalis* adherence. We hypothesized that the functionalization of BAR-encapsulated NPs with CafA would promote the adherence of BAR-encapsulated NPs to oral commensal streptococci such as *S. gordonii*, indicating future potential in targeting to *S. gordonii* harboring niches of the oral cavity. Since the *S. gordonii/P. gingivalis* interaction promotes supragingival *P. gingivalis* colonization, accumulation of BAR-encapsulated NPs and the localized, prolonged release of BAR from this niche may potently inhibit *S. gordonii/P. gingivalis* biofilm formation and reduce *P. gingivalis* colonization of the oral cavity.

The aliphatic polyester backbone of PLGA makes coupling ligands to the surfaces of the nanoparticles particularly challenging [44]. Previous studies have demonstrated that the incorporation of free ligands does not result in a stable protein presentation on the PLGA NP surface and that prior conjugation of ligands to lipids such as palmitic acid may help to achieve a stable protein coat [33,44]. To facilitate a sustained presentation of ligands, we conjugated CafA to palmitic acid prior to its addition during NP synthesis.

Following synthesis, we selected NPs with an intermediate density of surface ligands (20 µg/mg polymer) for further characterization and functional studies. Although in general, higher ligand density increases binding avidity via multivalent interactions, several studies have reported that NPs with intermediate ligand density exhibit higher binding relative to higher ligand densities [29,52,53]. Several explanations have been offered for this effect including steric interference; improper ligand orientation which may impede ligand function; or ligand overcrowding, leading to competition between ligands for the same receptor [29,52,53]. Additionally, these reports have suggested that increasing the ligand density beyond a certain threshold does not improve binding efficiency [54,55]. While NPs were formulated with an intermediate CafA concentration due to the feasibility of using a practical amount of peptide and the satisfactory release properties, these results demonstrate the in vitro effectiveness of the formulated NPs in functionality and efficacy. Future studies may focus on improving the efficacy of CafA-modified NPs by tuning ligand surface density to optimize binding avidity without impeding BAR release kinetics.

Surface modification can impact NP loading and modulate the release of encapsulants from NPs. Previous studies have found the encapsulation of a hydrophilic active agent, such as BAR peptide, to be challenging, due to its tendency to rapidly diffuse into the external aqueous phase during NP synthesis [56]. The use of amphipathic molecules has been shown to promote NP stabilization during synthesis and to reduce drug leakage into the external aqueous phase, enhancing encapsulation efficiency [44,57,58,59]. Results from our loading experiments performed with NPs modified with 20 µg CafA/mg polymer showed that CafA-modified and unmodified NPs encapsulate similar amounts of BAR (15.73 ± 1.90 and 16.95 ± 0.8 µg of BAR per mg of NP, respectively), with relatively high loading efficiency (37% and 39%), suggesting that surface modification at this density had minimal effect on BAR loading. While similar and high loading values were observed for BAR in both CafA-modified and unmodified NPs, approaches including altering polymer chemistry to improve polymer-protein affinity may be investigated to further increase encapsulation efficiency [29,60,61].

While both formulations exhibited high peptide loading, it is well known that polymeric NPs that encapsulate hydrophilic agents demonstrate high burst release due to the entrapment of the encapsulant in the form of small clusters on the surface or within the polymer matrix just below the particle surface, during synthesis [62]. Studies have shown that surface modification using amphipathic molecules slows the release of the encapsulant from polymeric NPs [29,44,57,58,62] due to increased hydrophobic stabilization and uniform dispersion of the encapsulant in the polymer matrix [59]. Our results were consistent with these findings in that unmodified BAR NPs demonstrated more rapid release corresponding to a burst (<50% peptide) within one hour of delivery. We attribute these findings to the release of BAR that is adsorbed at or just below the surface of the polymer matrix. After the first hour, minimal additional peptide was released from unmodified NPs. In comparison, CafA-modified NPs demonstrated a more gradual release of BAR peptide over 24 h, perhaps attributed to the potentially increased hydrophobic stabilization and more uniform dispersion of the peptide within the matrix, due to the presence of the CafA-palmitate conjugates on the NP surface. Cumulatively over 24 h, while 61% of the BAR peptide was released from unmodified NPs, CafA-modified NPs exhibited a near-complete release of the BAR peptide. This improved release was likely due to surface modification with hydrophilic CafA ligands, which facilitated the extraction of a higher amount of BAR, thereby promoting the complete release of the peptide payload from CafA-modified NPs.

In addition to high loading and favorable release kinetics, another desirable characteristic is NP retention at target sites for a prolonged duration. Given the site-specific nature of the periodontal disease, local application of therapeutic agents has been found to have better patient acceptance, reduced side-effects, and improved clinical outcomes. Although therapeutic agents applied directly to periodontal pockets are effective, they too get drained by the gingival crevicular fluid and saliva flow. Therefore, improving the binding characteristics of delivery vehicles can increase residence time at target sites and enhance NP efficacy. Furthermore, due to rising concerns related to antibiotic resistance and side-effects, there has been a tremendous interest in developing therapies targeting specific pathogens. Periodontal disease is a polymicrobial disease in which *P. gingivalis* plays a key role in inducing host dysbiosis and modulating immunity. Longitudinal metatranscriptomic analysis of the microbiome from stable to disease-progressing sites has shown that only *P. gingivalis* expresses virulence factors at healthy sites [63], suggesting that *P. gingivalis* initiates dysbiosis, fostering an environment that can contribute to the acceleration of disease processes [64]. Thus, while *P. gingivalis* is not essential for periodontal diseases, it is an important risk factor in periodontal diseases [64], and therefore targeting *P. gingivalis* is an effective strategy for developing targeted therapies for the prevention and treatment of periodontal diseases.

Here we targeted CafA-modified NPs to RPS-expressing bacteria such as *S. gordonii*, which promote *P. gingivalis* colonization. Our results demonstrate that CafA-modified NPs exhibit high binding and specificity to RPS-expressing commensal bacteria, relative to other bacteria including *S. mutans, P. gingivalis,* and *A. actinomycetemcomitans*, and remain bound to *S. gordonii*, while exerting functionality in a dual-species biofilm for up to 12 h.

While actively targeted NPs have been developed for use in tumor therapies and other applications, few studies have employed active targeting approaches for the treatment of periodontal diseases. Moreover, most actively targeted NPs developed for periodontal diseases are directed toward receptors on gingival epithelial cells [38]. To our knowledge, this is the first study to develop actively targeted NPs directed toward receptors on biofilm-forming bacterial surfaces, in which NPs are retained, release encapsulant that blocks specific pathogenic interactions, and limit the colonization of a specific pathogen that may potentially prevent host-microbiome dysbiosis.

Lastly, while CafA-modified NPs demonstrate significant promise in specific targeting, adherence, and retention to *S. gordonii*, we acknowledge that the in vivo delivery of CafA-modified NPs has challenges including the increased complexity of biofilms and dynamic flow conditions that may not be ideally reflected in vitro. To address this, future studies may focus on investigating the effects of salivary flow and consequent NP accumulation and residence time in the oral cavity by developing fluid dynamic models to assess the effects of fluid forces such as shear stress and saliva clearance on NP distribution and localization [63,64,65]. Additionally, future studies may focus on evaluating the release profile of BAR peptide from CafA-modified NPs in biorelevant conditions using a dialysis adapter-based USP apparatus 4 system in which issues relevant to very small NP sizes are overcome to maintain appropriate sink and agitation conditions that persist with typical dialysis methods [66]. Furthermore, the efficacy of CafA-modified NPs under dynamic conditions may be evaluated using multi-species biofilms in parallel flow chamber systems [67,68,69]. Lastly, murine models of periodontitis may also be used to study the localization, distribution, and corresponding effects of CafA-modified NPs after topical application to the oral cavity.

## 5. Conclusions

Overall, this study suggests that surface modification of NPs with specific biological ligands such as CafA can facilitate NP targeting and adherence to specific receptors on the surfaces of *S. gordonii* cells for a prolonged duration. Moreover, CafA-modified NPs release inhibitory concentrations of BAR peptide and potently inhibit *P. gingivalis* adherence to *S. gordonii* for a duration of time relevant to delivery in the oral cavity. CafA-modified NPs represent an efficacious vehicle for targeting BAR peptide to *P. gingivalis* preferred niches. Future experiments will focus on optimizing the surface density of the CafA and evaluating the functionality of these NPs. We anticipate that optimizing the surface density of CafA further will also enhance the retention, drug loading and release kinetics of modified NPs for durations relevant to twice-daily administration, increasing the overall efficacy of the NPs. In the long term, we hope to apply this work to incorporate CafA-modified NPs into oral gel formulations and test their effectiveness in pre-clinical animal studies.

## Figures and Tables

**Table 1:** Surface density of CafA input versus CafA conjugation concentration.

Input Concentration (µg CafA/mg NP)	Output Concentration (µg CafA/mg NP)	Conjugation Efficiency (%)
5	2.9 ± 0.1	58
10	7.9 ± 1.9	79
20	14.4 ± 2.6	72
40	25.4 ± 2.2	64
80	36.3 ± 3.5	45

**Table 2:** Percent inhibition of *P. gingivalis* adherence to *S. gordonii* at various time points.

Time Point (h)	Adherence Inhibition of *P. g*. (%)
*t* = 0	36.6 ± 8.8
*t* = 2	87.4 ± 6.9
*t* = 4	71.4 ± 7.2
*t* = 8	61.5 ± 2.7
*t* = 12	54.3 ± 5.6
